# IntAct: intra‐operative fluorescence angiography to prevent anastomotic leak in rectal cancer surgery: a randomized controlled trial

**DOI:** 10.1111/codi.14257

**Published:** 2018-06-08

**Authors:** G. Armstrong, J. Croft, N. Corrigan, J. M. Brown, V. Goh, P. Quirke, C. Hulme, D. Tolan, A. Kirby, R. Cahill, P. R. O'Connell, D. Miskovic, M. Coleman, D. Jayne

**Affiliations:** ^1^ St James’ University Hospital Leeds UK; ^2^ Clinical Trials Research Unit Leeds Institute of Clinical Trials Research University of Leeds Leeds UK; ^3^ School of Biomedical Engineering and Imaging Sciences King's College London and Honorary Consultant Radiologist Guy's and St Thomas’ Hospitals NHS Foundation Trust London UK; ^4^ University of Leeds Leeds UK; ^5^ Academic Unit of Health Economics Leeds Institute of Health Sciences University of Leeds Leeds UK; ^6^ Leeds Teaching Hospital Trust Leeds UK; ^7^ University College Dublin Dublin Ireland; ^8^ St Vincent's University Hospital Dublin Ireland; ^9^ St Mark's Hospital London UK; ^10^ Derriford Hospital Plymouth NHS Trust Plymouth UK; ^11^ Leeds Institute of Biological and Clinical Sciences St James's University Hospital Leeds UK

**Keywords:** Intra‐operative fluorescence angiography, resection, rectal cancer, anastomotic leak, randomized controlled trial

## Abstract

**Aim:**

Anastomotic leak (AL) is a major complication of rectal cancer surgery. Despite advances in surgical practice, the rates of AL have remained static, at around 10–15%. The aetiology of AL is multifactorial, but one of the most crucial risk factors, which is mostly under the control of the surgeon, is blood supply to the anastomosis. The MRC/NIHR IntAct study will determine whether assessment of anastomotic perfusion using a fluorescent dye (indocyanine green) and near‐infrared laparoscopy can minimize the rate of AL leak compared with conventional white‐light laparoscopy. Two mechanistic sub‐studies will explore the role of the rectal microbiome in AL and the predictive value of CT angiography/perfusion studies.

**Method:**

IntAct is a prospective, unblinded, parallel‐group, multicentre, European, randomized controlled trial comparing surgery with intra‐operative fluorescence angiography (IFA) against standard care (surgery with no IFA). The primary end‐point is rate of clinical AL at 90 days following surgery. Secondary end‐points include all AL (clinical and radiological), change in planned anastomosis, complications and re‐interventions, use of stoma, cost‐effectiveness of the intervention and quality of life. Patients should have a diagnosis of adenocarcinoma of the rectum suitable for potentially curative surgery by anterior resection. Over 3 years, 880 patients from 25 European centres will be recruited and followed up for 90 days.

**Discussion:**

IntAct will rigorously evaluate the use of IFA in rectal cancer surgery and explore the role of the microbiome in AL and the predictive value of preoperative CT angiography/perfusion scanning.

## Background

The most feared complication of rectal cancer surgery is anastomotic leak (AL), which is reported in 10–15% of patients 1, 2. AL has a negative impact on patient recovery and consumes a considerable amount of health resources for remedial interventions. It increases postoperative morbidity from ~20% to ~60% and mortality from < 5% to ~20%, and extends in‐patient stay by an additional 7 days on average 2, 3, 4. Patients who survive AL suffer long‐term consequences with reduced quality of life (QoL), high rates of wound complications, permanent stoma and increased risk of cancer recurrence 5, 6, 7, 8.

Several risk factors have been implicated in AL, including technical aspects of anastomosis construction (e.g. poor blood supply, inadequate tissue approximation and tension on the anastomosis) and patient risk factors associated with poor tissue healing (e.g. malnutrition and immunosuppression) 9, 10. Of all the factors that contribute to AL, probably the most crucial and the one that the surgeon has some influence over, is the blood supply to the anastomosis 11. Unfortunately, assessment of anastomotic perfusion is difficult during surgery, as demonstrated by the surgeon's inability to predict AL 12.

Intra‐operative fluorescence angiography (IFA) has been introduced to evaluate anastomotic blood supply, with promising early results. The technique involves intravenous administration of indocyanine green (ICG), which rapidly binds to plasma proteins and stays in the intravascular compartment. When irradiated with near‐infrared light (NIR), ICG fluorescence can be visualized on standard visual display units, providing an image of tissue perfusion.

Proof of concept has been established, but evidence is limited 13, 14, 15, 16, 17, 18, 19, 20. A multicentre, nonrandomized study – Perfusion Assessment in Laparoscopic Left Anterior Resection (PILLAR II) reported an AL rate of 1.4% in 139 patients available for analysis, which represents a reduction of eight‐ to nine‐fold in the documented leak rate of 12% following anterior resection (AR) 16. Degett *et al*.'s systematic review of 10 small nonrandomized trials (*n *=* *916) of IFA in colorectal anasotomosis surgery supports the early data of PILLAR II. The addition of IFA gave a pooled incidence of AL of 3.83% (95% CI: 2.64–5.02%) in colorectal resection surgery 17.

Although good surgical technique and optimal blood supply are paramount for anastomotic healing, there is increasing evidence to support a role for the gut microbiome 21. Using a rat model of AL, Shogan *et al*. 22 showed that anastomotic injury results in a change in anastomotic tissue‐associated microbiota, with upregulation of bacterial virulence‐associated pathways.

Neoadjuvant radiotherapy was used in the treatment of 37% of rectal cancers in the UK in 2014/2015 23. Although beneficial in reducing the risk of local cancer recurrence, preoperative radiotherapy increases the risk of AL 10, 24, 25, 26 through effects on the bowel microvasculature and possible alteration in the rectal microbiota 27, 28, 29, 30. Through the use of contrast‐enhanced CT angiography, it is possible to assess the anatomical variation in blood supply to the bowel, while perfusion CT can provide valuable quantitative information regarding bowel‐wall blood flow, blood volume and vascular leakage rate 31, 32, 33. The combination of CT angiography and perfusion CT may have a role in predicting AL and guiding operative decision‐making.

## Method

### Overall trial aims

IntAct will investigate the safety and efficacy of IFA in reducing AL rate following elective rectal cancer surgery. Two mechanistic sub‐studies will explore the role of the rectal microbiome in AL and the value of preoperative CT angiography and perfusion CT in predicting AL. The primary outcome measure will be clinical AL within 90 days of surgery. Secondary outcome measures will include all AL (clinical and radiological), complications and re‐interventions, change to anastomosis construction, use of stoma, as well as cost effectiveness and QoL outcomes.

### Trial sites and participating surgeons

IntAct will recruit from ≥ 25 European sites (Table 1). Participating sites must have access to an NIR laparoscopic or robotic system and be able to recruit a minimum of 12 patients per year. Surgeons must have experience of using IFA in three previous laparoscopic or robotic‐assisted ARs.

**Table 1:** IntAct anticipated participating sites

Principal investigator	Hospital	City	Country
Europe			
Professor Luigi Boni	Fondazione IRCCS Ca’ Granda Ospedale Maggiore Policlinico	Milan	Italy
Professor Frédéric Ris	Geneva University Hospitals	Geneva	Switzerland
Professor Ronan Cahill	Mater Misericordiae University Hospital, Dublin & Mater Private Hospital	Dublin	Ireland
Prof. Dr. med. Christoph Reißfelder	Chirurgische Klinik Universitätsmedizin	Mannheim	Germany
PD Dr. med. Christoph Holmer	Charité – Universitätsmedizin Berlin	Berlin	Germany
Professor Albert Wolthuis	University Hospital Leuven	Leuven	Belgium
Mr Roel Hompes	AMC Amsterdam	Amsterdam	The Netherlands
Dr Gabriele Barabino	CHU Sainte Etienne	Saint Etienne	France
Professor Pierre‐Emmanuel Colombo	ICM Val d'Aurelle Montpellier	Montpellier	France
Professor Giovanni Dapri	Saint‐Pierre University Hospital	Brussels	Belgium
UK			
Professor David Jayne	St James’ University Hospital	Leeds	
Mr Chris Cunningham	Churchill Hospital	Oxford	
Ms Deborah Nicol	Worcestershire Royal Hospital	Worcester	
Mr Mark Coleman	Derriford Hospital NHS Trust	Plymouth	
Mr Manish Chand	University College Hospital (UCLH)	London	
Mr James Horwood	University Hospital of Wales	Cardiff	
Mr Peter Coyne	Royal Victoria Infirmary	Newcastle	
Mr Mohamed Adhnan Thaha	The Royal London Hospital	London	
Professor Tim Rockall	The Royal Surrey County Hospital	Surrey	
Mr Charles Evans	University Hospital Coventry	Coventry	
Mr Athur Harikrishnan	Northern General Hospital	Sheffield	
Mr John Griffith	Bradford Royal Infirmary	Bradford	
Mr Ioannis Peristerakis	Royal Preston Hospital	Preston	
Mr Henry Tilney	Frimley Park Hospital	Surrey	
Mr Danilo Miskovic	St Mark's Hospital	London	
Mr Paul Mackey	Musgrove Park Hospital	Taunton	
Mr Stephen Dalton	Royal United Hospital Bath	Bath	
Mr Praminthra Chitsabesan	York Teaching Hospital	York	

Information correct at time of publication. Participating site and/or the designated principal investigator may change throughout the course of the trial.

At least 25 sites across the UK and Europe will participate in IntAct. Site are expected to recruit a minimum of 12 patients per year. Additional sites will be added in due course.

### Trial population

Patients will be over 18 years of age with a diagnosis of adenocarcinoma of the rectum (defined as a lower margin ≤ 15 cm from the anal verge on endoscopic or radiological assessment) that is suitable for elective curative resection by high or low anterior resection. The procedure can be performed either laparoscopically or with robotic assistance.

Patients will be ineligible if not undergoing primary colorectal/anal anastomosis or if undergoing synchronous colonic resection. Patients with recurrent or locally advanced rectal cancer requiring extended or multivisceral excision will be excluded. Other exclusion criteria include: coexistent colorectal pathology (e.g. inflammatory bowel disease); previous remote pelvic radiotherapy (e.g. previous prostate cancer treated with radiotherapy); antibiotic medication within 8 weeks of randomization; pregnancy; allergy to iodine or ICG; and hepatic or renal dysfunction.

### Trial design

**Figure 1:**
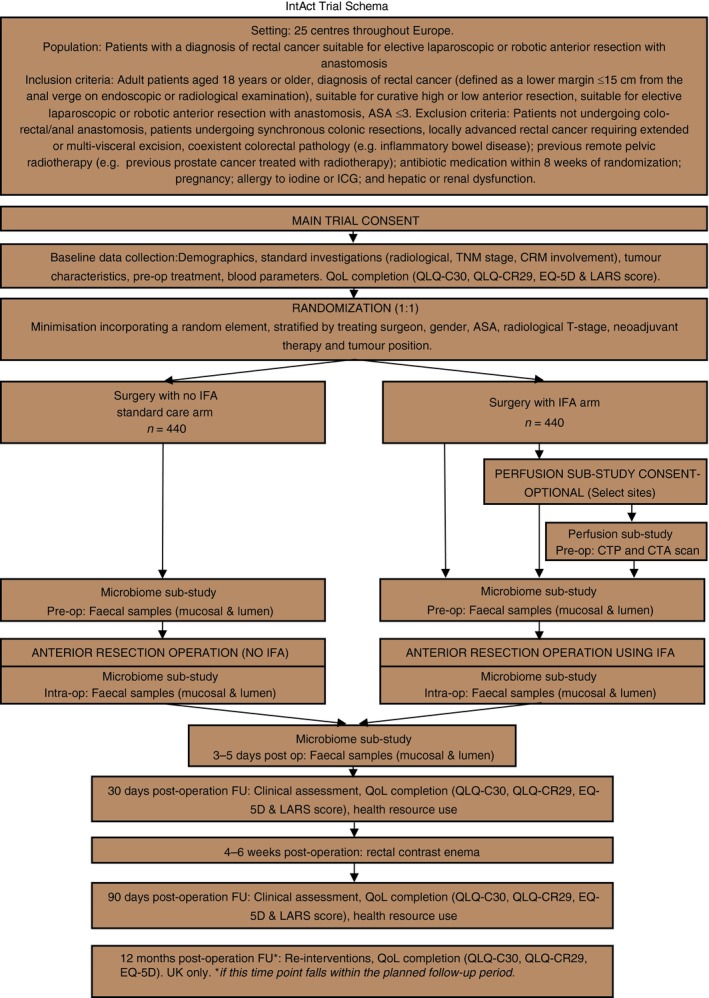
IntAct trial Schema. ASA, American Society of Anesthesiologists; CRM, circumferential resection margin; CTA, CT angiography; CTp, perfusion CT; FU, follow‐up; ICG, indocyanine green; IFA, intra‐operative fluorescence angiography; Intra‐op, intra‐operative; LARS, low anterior resection syndrome; QoL, quality of life; Pre‐op, preoperative; post op, postoperative.

IntAct is a prospective, unblinded, parallel‐group, multicentre, European, randomized controlled trial comparing surgery with IFA against standard care (surgery with no IFA). The primary outcome is the difference in clinical AL rate at 90 days postsurgery between the two groups. The Clinical Trials Research Unit (CTRU) at the University of Leeds will co‐ordinate the trial. The trial will run for 3 years and all patients will be followed‐up for 90 days from the date of surgery. UK patients recruited within the first 2 years of enrolment will provide QoL and health resource utilization data at 1 year (Fig. 1).

### Sample size

A total of 880 patients will be recruited over 36 months and randomized on a 1:1 basis to receive surgery, either with or without IFA. A computer‐generated minimization program incorporating a random element will be used to ensure that treatment groups are well balanced (Fig. 1).

A formal interim analysis will be conducted once primary end‐point data are available for 554 patients. In the UK, the first 200 patients recruited will take part in the microbiome sub‐study, and 75 patients randomized to IFA will take part in the optional perfusion sub‐study.

### Randomization timing

Randomization will be performed at the time of obtaining informed consent. This will be as close to date of surgery as is feasibly possible and no more than 28 days prior to surgery.

### Interventions

#### Standard care arm (no IFA)

Laparoscopic or robot‐assisted AR (high or low) will be performed as per surgeon preference. Perfusion assessment will be performed with white light (WL) only.

#### IFA arm

For participants randomized to surgery with IFA, AR (AR) will be performed according to the surgeon's usual technique, using either a laparoscopic or a robotic approach.

Two IFA assessments are required, each involving an intravenous bolus of 0.1 mg/kg of ICG. The first assessment will be performed after rectal mobilization but before bowel resection. Under WL laparoscopy, the point of planned bowel transection will be marked. Using either an intra‐ or extracorporeal IFA technique, the time to first fluorescence and any change in the planned bowel transection point following IFA will be recorded. The fluorescence intensity will be graded using a semi‐quantifiable scale. The second assessment will be performed after construction of the anastomosis. This can be either an intracorporeal or endoluminal assessment following ICG administration, with time to fluorescence and fluorescence intensity recorded. One additional IFA assessment is permitted at the operating surgeon's discretion.

#### Sub‐study interventions

The first 200 UK patients recruited to either intervention group will provide rectal luminal and mucosal faecal samples at baseline, intra‐operatively and 3–5 days postsurgery. Rectal bacteria will be classified into taxonomic groups by 16S ribosomal RNA (rRNA) gene analysis and assessed for collagenase activity on solid agar.

At selected UK centres, the first 75 patients recruited to the IFA intervention can participate in the optional contrast‐enhanced CT angiography and perfusion CT sub‐study. The perfusion CT scan will be centred on the planned site of rectal anastomosis and performed within 14 days of planned surgery. This will allow the attenuation over time to be plotted for the rectal wall and for regional blood flow, blood volume and permeability surface area product to be derived. Perfusion CT will be immediately followed by the CT angiography scan to allow anatomical evaluation of the vasculature with image reconstructions in the sagittal and coronal planes and three‐dimensional (3D) volume rendering.

### Postoperative care

Postoperative care will be as per institutional protocol. All participants will receive clinical assessment at 30 and 90 days postsurgery and will undergo a rectal contrast enema around 4–6 weeks postsurgery to detect radiological evidence of AL (unless AL has been confirmed by alternative means).

### Patient completed questionnaires

Patient completed questionnaires measuring QoL (EQ‐5D, EORTC QLQ‐C30, EORTC QLQ‐CR29 and LARS), and Health Resource Use questionnaires, will be completed at baseline, and 30 and 90 days postsurgery. There will be an additional QoL questionnaire pack (EQ‐5D, EORTC QLQ‐C30, EORTC QLQ‐CR29) posted to UK patients at 1 year postsurgery if this time point falls before the end of the planned follow‐up period (i.e. 90 days following the last patient's operation) (Table 2).

**Table 2:** Schedule of events

	Event	Baseline/Pre‐op	Surgery	3–5 days postop	30 days postop	4–6 weeks postop	90 days postop	1 year postop (UK only)
Clinical assessments/investigations	Clinical examination	✓			✓		✓	
	Pre‐operative bloods	✓						
	Operative details		✓					
	Complications		✓		✓		✓	
	Trial consent	✓						
	Microbiome sub‐study (faecal samples)	✓	✓	✓				
	Perfusion sub‐study (CTP and CTA) – OPTIONAL	✓						
	Rectal contrast enema scan					✓		
Data collection time points	Eligibility CRF	✓						
	Baseline CRF	✓						
	Operative CRF		✓					
	30 days postsurgery f/up CRF				✓			
	90 days postsurgery f/up CRF						✓	
	1 year postsurgery f/up CRF							✓^a^
Participant completed questionnaires	EQ‐5D‐5L	✓			✓		✓	✓^a^
	EORTC QLQ‐C30 and QLQ‐CR29	✓			✓		✓	✓^a^
	LARS	✓			✓		✓	
	Resource use (UK sites only)	✓			✓		✓	

CRF, case report form; CTA, CT angiography; CTp, perfusion CT; f/up, follow‐up; LARS, low anterior resection syndrome; postop, postoperative; Pre‐op, preoperative.

a Only required if this time point falls before the end of the planned follow‐up period (i.e. 90 days following the last participant's operation).

### Data collection and management

Participating sites will record patient data on trial‐specific case report forms (CRFs). Data will be collected at baseline, intra‐operatively and at clinical assessment on days 30 and 90 postsurgery. Hospital readmission details from medical notes will be collected for UK patients only at 1 year postsurgery, if this time point falls before the end of the planned follow‐up period.

Participant completed data will be collected as per Table 2. The CTRU will provide overall data and trial management.

### Outcomes

The primary end‐point of the main trial is clinical AL rate within 90 days of surgery. AL is defined, according to the International Study Group definition 34, as a confirmed defect of the intestinal wall at the anastomotic site (including suture and staple lines of neorectal reservoirs) leading to a communication between the intra‐ and extraluminal compartments that has an impact on patient management. An abscess or collection of fluid in close proximity to the anastomosis will be deemed as an AL. This equates to Grades B and C in the International Study Group definition.

The secondary end‐points are AL (all grades), change in intra‐operative decision‐making (including change to planned anastomosis), use of stoma, complications and re‐interventions, QoL outcomes measures, low anterior resection syndrome (LARS) scores and health resource utilization measures.

The CT perfusion sub‐study will examine the effects of radiotherapy and anatomical variance in vascular anatomy on rectal blood flow and their relationship to AL. It will also provide additional data to aid interpretation of results in cases where poor IFA perfusion is observed. The microbiome sub‐study will provide data on the taxonomic classification and collagenase activity of rectal microorganisms, the effect on mechanical bowel preparation and surgery on the microbiome and will allow correlation with the rate of AL in the study population.

### Quality of life

Trial participants will complete several questionnaires to capture health status (EQ‐5D‐5L) and QoL (QLQ‐C30 and QLQ‐CR29) at 30 and 90 days postsurgery (and at day 365 where permitted). Patients without a defunctioning stoma will provide information on bowel function and LARS.

### Health economic assessment

Analyses will report the differences in the cost of health and social care service utilization between groups and the incremental cost‐effectiveness ratios using both the same primary outcome as the trial and quality adjusted life years. Resource use will be collected through investigator and participant completed forms at the 30‐ and 90‐day postsurgery assessment. Unit costs for resources will be obtained from national sources, such as the NHS Reference cost database. The nonparametric bootstrap method will be used to produce a within‐trial probabilistic sensitivity analysis of the incremental cost‐effectiveness ratio. IntAct will present the expected incremental cost‐effectiveness ratio, the scatterplot on the cost‐effectiveness plane, the 95% cost‐effectiveness ellipse and the cost‐effectiveness acceptability curve 35.

### Statistical methods

A sample size of 880 patients is required to show a reduction in clinical AL rate from 12.0% to 6.0% at a two‐sided 5% level of significance with 80% power, allowing for a formal interim analysis (details below) and a 10% drop‐out rate.

The rate of clinical AL in each trial arm will be summarized according to trial arm alongside measures of uncertainty. The primary analysis will compare AL rates between the arms using multilevel logistic regression incorporating random effects with respect to surgeon and adjusting for the stratification factors. This approach will be used to test the two‐sided hypothesis that the AL rate is equal in both arms (i.e. OR = 1), considering the 95% CI and the *P*‐value yielded by a Wald test of the treatment allocation regression coefficient.

The formal interim analysis will be conducted on unblinded data once primary end‐point data are available for 554 participants. At the interim analysis, a value of *P *<* *0.0146 will be considered to be sufficiently strong evidence of efficacy for early stopping. At the final primary analysis, a value of *P *<* *0.0456 will be considered as ‘significant’ in order to maintain the overall type I error rate (as per O'Brien and Fleming) 36. The timing of the interim analysis was chosen such that it occurs as soon as possible after the sub‐studies have been completed, whilst maintaining acceptable operating characteristics.

### Safety evaluation and reporting of adverse events

For the purpose of this surgical trial, adverse events will be termed as complications. A complication is defined as an untoward medical event in a participant which has a causal relationship to the trial. The trial includes the surgical intervention and any trial‐specific interventions. Information on all complications will be collected, this includes information volunteered by the participant or discovered by the investigator.

### Trial organization, administration and governance

IntAct is funded by the Efficacy and Mechanism Evaluation (EME) Programme, an MRC and NIHR partnership (Grant Reference: 14/150/62). The trial sponsor is the University of Leeds. IntAct will be overseen by an independent Data Monitoring and Ethics Committee (DMEC) and Trial Steering Committee. The study has been designed with input from public and patient groups and is supported by Bowel Cancer UK.

### Ethical considerations

The trial will be conducted in accordance with the principles of Good Clinical Practice (GCP) in clinical trials, the NHS Research Governance Framework and through adherence to CTRU Standard Operating Procedures (SOPs). The trial will operate using the recommendations guiding physicians in biomedical research involving human subjects adopted by the 18th World Medical Assembly, Helsinki, Finland, 1964, amended at the 64th World Medical Association General Assembly, Fortaleza, Brazil, October 2013 37.

IntAct has been granted ethical approval by the UK Health Research Authority (HRA) Research Ethics Committee (REC).

## Discussion

There has been no advance in eliminating the most feared complication of gastrointestinal surgery – AL – in the past 50 years. This proposal evaluates a new technology – IFA – which, for the first time, allows surgeons to assess intra‐operative anastomotic perfusion easily, could help to reduce the incidence of AL significantly. The incorporation of two sub‐studies evaluating rectal blood supply and perfusion in patients with and without neoadjuvant chemo/radiotherapy, and the role of the rectal microbiome in AL, adds exciting dimensions that will further inform our understanding of the mechanisms underlying AL.

Although previous studies have suggested that IFA can reduce the risk of AL, the scientific rigour of these evaluations was limited. PILLAR II was a non‐randomized, observational study including a mixed cohort of patients with malignant and benign colorectal conditions and restricted to the USA. No assessment was made of QoL or cost‐effectiveness, and its generalizability is questionable. Other studies have consisted mainly of small observational series and are subject to selection and reporting biases 13, 15, 16, 17, 19, 33.

As yet, no randomized controlled trial of IFA as a method of reducing AL in rectal cancer surgery has been reported. The PILLAR III study (ClinicalTrials.gov registry identifier NCT02205307) was a multicentre randomized controlled trial of IFA in the USA and has terminated early. PILLAR III recruited 330 of a planned 550–900 patients undergoing AR (≥ 10 cm from the anal verge) before May 2017 38. This industry‐funded trial used clinical AL as the primary end‐point and aimed to assess the efficacy of IFA in preventing AL. IntAct differs in design to PILLAR III in assessing both clinical and radiological AL and with the inclusion of cost‐effectiveness as trial end‐points. It investigates a European surgical population where practice can differ from that in the USA. Importantly, IntAct includes two exciting sub‐studies that may help in understanding the mechansims underlying AL.

If IntAct confirms the efficacy of IFA in minimizing AL it will have widespread implications, not just for rectal cancer surgery but potentially for any surgery involving an anastomosis. AL is a burden on valuable healthcare resources; the average cost of AL following AR is £17 220 39. It is one of the leading causes of death following rectal cancer surgery, with around 20–30% of all 30‐day mortality being directly attributed to AL 7, 8. In those patients who survive AL, it is a cause of long‐term morbidity, associated with poor bowel function, reduced QoL, increased risk of cancer recurrence and high rates of permanent stoma 5, 6, 9, 40.

If IntAct confirms the hypothesis that IFA reduces the rate of AL, it will inform the uptake and provision of the technology, with potential cost savings for healthcare providers and safer surgery for patients.

## Disclaimer

The views expressed are those of the author(s) and not necessarily those of the NHS, the NIHR or the Department of Health and Social Care.

## Author contributions

All authors contributed to development of the trial and writing of the manuscript.

## Conflicts of interest

The authors declare that they have no conflict of interest.
